# Exploring the effects of a wearable biocueing app (Sense-IT) as an addition to aggression regulation therapy in forensic psychiatric outpatients

**DOI:** 10.3389/fpsyg.2023.983286

**Published:** 2023-03-10

**Authors:** Janna F. ter Harmsel, Matthijs L. Noordzij, Thimo M. van der Pol, Lise T. A. Swinkels, Anna E. Goudriaan, Arne Popma

**Affiliations:** ^1^Forensic Mental Healthcare, Inforsa, Amsterdam, Netherlands; ^2^Department of Child and Adolescent Psychiatry and Psychology, Amsterdam Public Health Research Institute, Amsterdam UMC, Vrije Universiteit Amsterdam, Amsterdam, Netherlands; ^3^Department of Psychology, Health and Technology, University of Twente, Enschede, Netherlands; ^4^Department of Research and Quality of Care, Arkin Mental Health Institute, Amsterdam, Netherlands; ^5^Department of Psychiatry, Amsterdam Public Health Research Institute, Amsterdam UMC, University of Amsterdam, Amsterdam, Netherlands; ^6^Amsterdam Institute for Addiction Research, Amsterdam, Netherlands

**Keywords:** biocueing, biofeedback, aggression, emotion regulation, forensic psychiatry, wearable technology, mHealth

## Abstract

**Objective:**

Preventing and reducing violence is of high importance for both individuals and society. However, the overall efficacy of current treatment interventions aimed at reducing aggressive behavior is limited. New technological-based interventions may enhance treatment outcomes, for instance by facilitating out-of-session practice and providing just-in-time support. Therefore, the aim of this study was to assess the effects of the Sense-IT biocueing app as an addition to aggression regulation therapy (ART) on interoceptive awareness, emotion regulation, and aggressive behavior among forensic outpatients.

**Methods:**

A combination of methods was used. Quantitatively, a pretest-posttest design was applied to explore group changes in aggression, emotion regulation, and anger bodily sensations associated with the combination of biocueing intervention and ART. Measures were assessed at pretest, after 4 weeks posttest, and after one-month follow-up. During the 4 weeks, a single-case experimental ABA design was applied for each participant. Biocueing was added in the intervention phase. During all phases anger, aggressive thoughts, aggressive behavior, behavioral control, and physical tension were assessed twice a day, and heart rate was measured continuously. Qualitative information regarding interoceptive awareness, coping, and aggression was collected at posttest. 25 forensic outpatients participated.

**Results:**

A significant decrease in self-reported aggression was found between pre- and posttest. Furthermore, three-quarters of participants reported increased interoceptive awareness associated with the biocueing intervention. However, the repeated ambulatory measurements of the single-case experimental designs (SCEDs) did not indicate a clear effect favoring the addition of biocueing. On group level, no significant effects were found. On the individual level, effects favoring the intervention were only found for two participants. Overall, effect sizes were small.

**Conclusion:**

Biocueing seems a helpful addition to increase interoceptive awareness among forensic outpatients. However, not all patients benefit from the current intervention and, more specifically, from its behavioral support component aimed at enhancing emotion regulation. Future studies should therefore focus on increasing usability, tailoring the intervention to individual needs, and on integration into therapy. Individual characteristics associated with effective support by a biocueing intervention should be further investigated, as the use of personalized and technological-based treatment interventions is expected to increase in the coming years.

## 1. Introduction

Reducing aggressive behavior and criminal recidivism is an important goal in forensic psychiatry. For this purpose, several treatment interventions have been developed over the last decades. Most of these interventions are based on cognitive behavioral therapeutic principles and share elements with Aggression Replacement Training (Goldstein et al., 1998): a treatment program in which behavioral, affective, and cognitive components are combined to improve aggression regulation. However, although risk reductions in violent recidivism have been reported in several studies (Henwood et al., 2015), the overall efficacy of these treatment interventions aimed at reducing aggressive behavior has been found to be limited (Brännström et al., 2016; McIntosh et al., 2021). Since risk reductions are more pronounced among treatment completers (Henwood et al., 2015; Brännström et al., 2016), part of the limited effectivity of the current programs is probably related to low treatment adherence. Important to note is that low adherence may not only result in dropout but might also constrain the transfer of therapeutic skills into daily practice by impairing the completion of out-of-session assignments (Fletcher et al., 2011; Kazantzis et al., 2016). Furthermore, by focusing on achievement of cognitive control over emotional responses, current treatment programs might pay insufficient attention to other prerequisites of adequate anger regulation, such as awareness and recognition of psychophysiological signals associated with aggression and other challenging behaviors (McDonnell et al., 2015; Price and Hooven, 2018; Bellemans et al., 2019).

Over the last years, the number of studies focusing on the psychophysiological correlates of antisocial spectrum behavior and aggression has increased (Portnoy and Farrington, 2015; Blankenstein et al., 2021; De Looff et al., 2021; Blankenstein et al., 2022). In aggression research, psychophysiological measures such as heart rate (HR), skin conductance level (SCL), and heart rate variability (HRV) are used as indicators of, respectively, the general activity of the autonomic nervous system (ANS) and its two branches: the accelerating sympathetic nervous system (SNS) and the inhibitory parasympathetic nervous system (PNS; Branje and Koot, 2018). To understand the underlying mechanisms of aggressive behavior, ANS patterns of patients with aggression regulation difficulties have been compared to those of healthy controls, both at rest as well as in response to arousal-inducing events (i.e., reactivity measures; Blankenstein et al., 2022). Recent meta-analyses demonstrated that lower HR at rest has most consistently been found to be positively related to antisocial behavior in general and proactive aggression in particular, although the overall effect size is small (Portnoy and Farrington, 2015; De Looff et al., 2021). The research findings for reactivity measures are mixed. Regarding overall ANS reactivity, previous studies have shown increases in HR reactivity in response to emotional stimuli (Lorber, 2004; Ortiz and Raine, 2004) and provocation, associated with reactive aggression (Crozier et al., 2008). Other research results demonstrated blunted HR reactivity, suggesting diminished sensitivity to stressors such as threat or punishment associated with proactive aggression (Van Goozen et al., 2007). Furthermore, there is some evidence that reactive aggression is related to heightened SNS reactivity (Murray-Close et al., 2017; Armstrong et al., 2019; Thomson et al., 2021) and proactive aggression to blunted SNS and PNS reactivity (Patrick, 2014; Moore et al., 2018; Armstrong et al., 2019; Thomson et al., 2021). However, null findings for one or both associations have also been reported (Centifanti et al., 2013; Wagner and Abaied, 2015; Zijlmans et al., 2021; Ter Harmsel et al., 2022b). With researchers stressing the importance of studying the interaction between SNS and PNS to understand proactive and reactive aggression, instead of hypo- or hyperreactivity of the subsystems alone (Branje and Koot, 2018; Moore et al., 2018; Puhalla and McCloskey, 2020), the psychophysiological reactivity results remain largely inconclusive to date.

Psychophysiological measures are not only used to understand aggressive behavior but can also be used to predict aggressive incidents in real life. For a long time most studies aimed at identifying these physiological biomarkers were conducted in laboratory settings (Adams et al., 2017). However, in recent years first pioneering studies have been conducted in clinical settings, among inpatients with aggressive behavior. In a naturalistic study among patients with intellectual disabilities and behavioral problems, non-linear fluctuations in HRV (i.e., decreases in the first levels of increasing tension and a sudden increase when reaching extreme agitation) were found prior to outbursts (Palix et al., 2017). Studies among children and adolescents with autism spectrum disorders demonstrated that challenging or aggressive behaviors could be predicted approximately 1 min before occurrence using biosensor HR data of the preceding minutes (Goodwin et al., 2019; Nuske et al., 2019). Furthermore, aggressive incidents among forensic inpatients turned out to be preceded by significant increases in HR and SCL up to 20 min before manifestation (De Looff et al., 2019).

Some of the aforementioned challenges in treatment of forensic outpatients with aggressive behavior, such as the difficulties in recognizing physiological signals that precede aggressive incidents and the limitations in out-of-session practice, might be addressed by implementing the psychophysiological research results facilitated by the fast developments in e- and m-health technology. New interventions, such as serious gaming (Smeijers and Koole, 2019), virtual reality therapy (Klein Tuente et al., 2020), and mobile biofeedback or biocueing apps (Mackintosh et al., 2017), create opportunities to increase treatment adherence by enhancing motivation and by lowering barriers for out-of-session practice. Whereas serious gaming and virtual reality therapy are delivered on-site, at home, or in a clinical setting, biocueing could provide the patient with just-in-time behavioral support by real-time measurement in everyday life (Riley et al., 2015; Nahum-Shani et al., 2018).

This new intervention, biocueing, can be considered a derivate of traditional biofeedback, in which users are provided with real-time physiological information and trained to influence physiological parameters, such as HRV (Lehrer, 2013) or cardiac coherence (McCraty and Zayas, 2014), by consciously alternating their (breathing) responses to the given feedback. In the process of biocueing wearable and mobile devices are used to collect and display the physiological biomarkers to the user in a direct way (Ter Harmsel J. F. et al., 2021). In contrast with traditional biofeedback, biocueing is more focused on aiding and enhancing momentary awareness of physiological sensations (i.e., interoceptive awareness) and internal emotional experiences (i.e., emotional awareness), and to a lesser extent on deliberate training of regulation techniques. In biocueing, the training component is restricted to the moments when physiological tension elevates and the user receives a just-in-time message encouraging the use of adequate coping strategies (Nahum-Shani et al., 2018). Both components of biocueing interventions – increasing interoceptive awareness and delivering just-in-time behavioral support – may be helpful to reduce and prevent aggressive incidents among forensics outpatients (Cornet et al., 2017; Ter Harmsel J. F. et al., 2021).

Given the potential of biocueing for the forensic population, we investigated the acceptability, usability, and clinical changes associated with the use of an earlier version of the Sense-IT biocueing app (Derks et al., 2019) in a two-week evaluation study among forensic outpatients (Ter Harmsel A. et al., 2021). Using the feedback of these end-users, a new version of the app was developed. The aim of the current study was to assess the effects of the new version of the Sense-IT biocueing app as an addition to aggression regulation therapy (ART) on interoceptive awareness, emotion regulation, and aggressive behavior among forensic outpatients. Quantitatively, we expected that the combination of biocueing intervention and ART would be associated with positive group changes between pretest, posttest, and follow-up on measures of aggression, emotion regulation and insight in anger bodily sensations (pretest-posttest design). Furthermore, we hypothesized group and individual increases in behavioral control and decreases in aggressive behavior as well as changes in exploratory measures anger, aggressive thoughts, physical tension and HR favoring the biocueing intervention phase (single-case experimental designs, SCEDs). For the qualitative part of this study, perceived effectivity would be indicated by patient-reported increases in interoceptive awareness, use of coping strategies, and prevention of aggressive incidents associated with the use of the Sense-IT app.

## 2. Materials and methods

### 2.1. Design

In this study, we used a combination of methods to answer the research question. Forensic outpatients receiving ART were invited to use the Sense-IT app (Derks et al., 2017, 2019) for 4 weeks. A pretest-posttest design was applied to examine changes on group level. Quantitative data were administered at the start (T0), after the 4 weeks (T1), and after one-month follow-up (T2). During the 4 week period a single-case experimental ABA design was applied for each participant, in which a baseline phase (A_1_), was followed by an intervention phase (B) and a follow-up phase (A_2_). In the two-week intervention phase, biocueing was added. Initially, we planned to randomize the start of the B-phase to either 5, 7, or 9 days after the start of phase A_1_ for each group of three participants. However, since this procedure could not be aligned to the routines and schedules of potential participants, we had to let go of this multiple baseline aspect of the design. During all phases, the emotional state of the participants was assessed twice a day and HR was continuously measured. Qualitative data was collected at T1 *via* semi-structured interviews, enabling us to obtain a deeper understanding of patients’ experiences concerning the effectivity of the Sense-IT. The study protocol and subsequent amendments were approved by the Medical Ethical Committee of Amsterdam University Medical Centre, Vrije Universiteit, Netherlands (NL63911.029.17). The study was registered in Netherlands Trial Register (NL8206).

### 2.2. Participants

**Figure 1:**
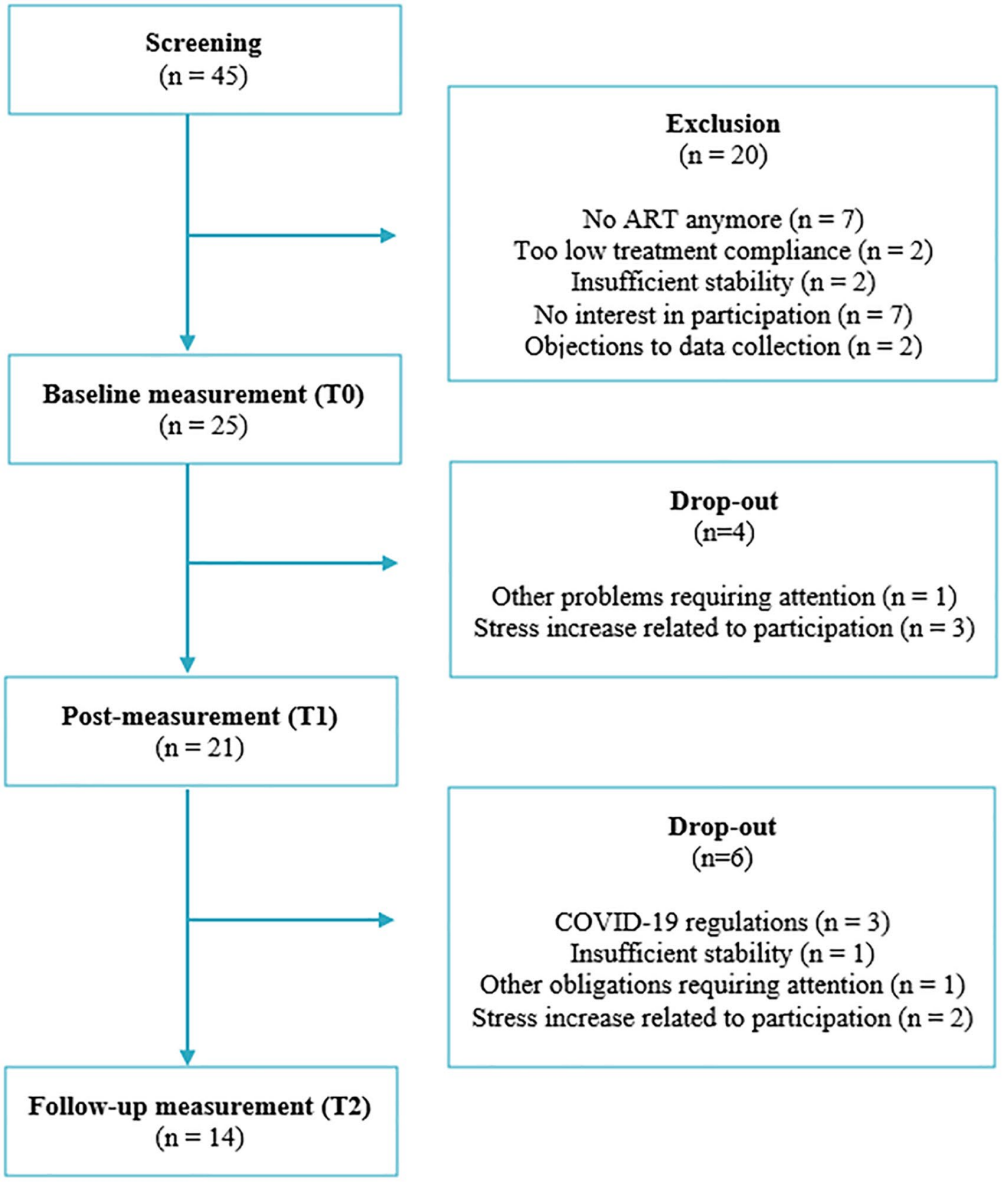
Flow chart of recruitment and participation.

Forensic outpatients, receiving ART at Inforsa, a forensic mental healthcare facility in the Netherlands, were recruited for participation in this study from 2020 to 2022. Potential participants were screened for eligibility by a research associate, consulting the patients’ therapist. The eligibility criteria included: (1) a proven lack of anger management skills, indicated by either a recently committed violent crime and/or a high risk of committing one; (2) assignment to individual outpatient ART after multidisciplinary consultation; (3) basic understanding of mobile applications; and (4) an age of 16 years or above. The exclusion criteria included: (1) acute manic or psychotic symptoms; (2) current high risk of suicide; (3) severe addiction problems or other severe conditions requiring immediate intervention or hospitalization; and (4) insufficient understanding of the Dutch language. The first two inclusion criteria were assessed by checking the committed index defense (if applicable) and the clinical decisions recorded in the electronic patient file. The first three exclusion criteria were assessed by consulting the patients’ therapist, using cut-off scores on the corresponding items of the Health of the Nations Outcome Scales (HoNOS; Wing et al., 1998). After screening and presenting the research project to eligible patients, 25 patients were willing to participate and enrolled in the study. Reasons for drop-out were: premature study termination due to COVID-19 regulations, reported stress increase related to participation in the study, other problems or obligations requiring attention, and insufficient stability. An outline of the recruitment and participation flow is displayed in Figure 1.

### 2.3. Procedure

Eligible patients expressing interest in the research project received a face-to-face appointment in the presence of their therapist to discuss study participation. The research associate provided the patient with a brief oral description and full written information about the study. The voluntary nature and the absence of any negative consequences refusing participation were emphasized. When the patient expressed willingness to participate, the next appointment was planned after at least 7 days, providing enough time for consideration. In this appointment written informed consent was obtained and baseline measurement (T0) was administered, which lasted approximately 60 min. After completion of the questionnaires, participants were provided with a smartwatch and mobile phone with the Sense-IT app. Participants were shown how to use the devices and were advised on charging and using the system safely. They also received a user manual. Participants used the devices independently during the following 4 weeks. They were encouraged to call the research associates if any problem occurred. During these weeks, the research associate met with the participants twice; once to start the intervention phase (B) and once to start the follow-up phase (A_2_). In these short appointments questions were answered, if applicable, and participants were reminded to answer the daily questions. After these 4 weeks, another 60 min assessment (T1) was planned, in which both qualitative and quantitative measures were administered. One month after T1-assessment, a 30 min follow-up assessment (T2) was scheduled. An overview of outcomes and their moment of assessment is presented in Table 1.

**Table 1:** Overview of outcomes, measures, and moment of assessment.

Outcome	Measure	T0	Tx (during SCED)	T1	T2
Aggressive behavior	AQ-SF	+		+	+
	MOAS	+		+	+
HR measures	Biosensor		+		
Emotional state	EMA		+		
Emotion regulation	DERS	+		+	+
Anger regulation	STAXI-2	+			
Anger bodily sensations	ABSQ	+		+	+
Aggression type	RPQ	+			
Psychopathy	YPI-s	+			
Judicial history	File information	+			
Demographic information	DQ	+			
Evaluation of the Sense-IT	Qualitative interview			+	+
System usability	SUS			+	

AQ-SF, Aggression Questionnaire-Short Form; MOAS, Modified Overt Aggression Scale; HR, Heart Rate; EMA, Ecological Momentary Assessment; DERS, Difficulties in Emotion Regulation Scale; STAXI-2, State–Trait Anger Expression Inventory; ABSQ, Anger Bodily Sensations Questionnaire; RPQ, Reactive Proactive Questionnaire; YPI-s, Youth Psychopathic Traits Inventory-Short Version; DQ, Demographic Questionnaire; SUS, System Usability Scale.

### 2.4. Materials

Below, we will first introduce the studied intervention, the Sense-IT app. Next, we describe the quantitative measures used in the pretest-posttest design (change and descriptive measures) and the SCEDs (self-report and physiological measures). Finally, we describe the qualitative measures.

#### 2.4.1. Sense-It

**Figure 2:**
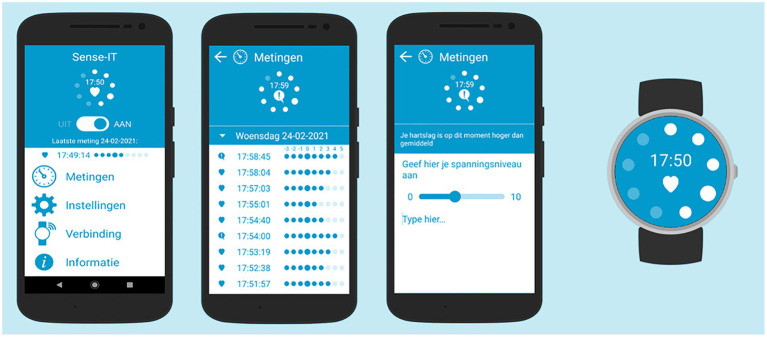
Screenshots of the Sense-IT app (version 2.57) with the main screen (presenting four menus: measurements, settings, connectivity, and information), measurement screen (presenting HR levels), notes screen (presenting default message, question to rate subjective level of arousal, and space for personal notes) and one of the watch faces.

The newly developed version of the Sense-IT app, version 2.57 (with some minor bug fixes), was preinstalled on all smartwatches and mobile phones before distribution. The Sense-IT app was originally developed by the University of Twente and Scelta, an expert center for psychiatric patients with personality disorders (Derks et al., 2017, 2019) and modified to fit the needs of forensic outpatients assessed in an earlier study (Ter Harmsel A. et al., 2021). In the current study, we replaced the Ticwatch E, which we also used in our previous study, with the Ticwatch E2 (Mobvoi, Ltd). Compared to its predecessor the E2 is sleeker, more sophisticated, and has a slightly longer battery life. Connection with the mobile phone, the Moto C Plus or the Moto E6 Play (Google, LLC), was established *via* Bluetooth. The Sense-IT system reads the physiological data measured by the photoplethysmography (PPG) sensor and stores the data in a local database on the smartphone itself. The build-in algorithm compares the current HR to the user’s mean HR at baseline and calculates a level between −3 and 5 using the standard deviation of the baseline measurement. In the current study, we further refined the baseline measurement procedure. Ultimately, baseline measurement was performed during T0, included at least 1 min of sitting in quiet, 1 min of social interaction, and 1 min of walking activity to account for sufficient variation, and lasted until the PPG sensor received 500 reliable HR measures. Our starting values for HR and heart rate variance thresholds were in line with published norms indicating a mean HR around 80 (SD ~ 7; Umetani et al., 1998). More information on the baseline procedure can be found in the Supplementary material. After baseline measurement, the real-time HR level is visually displayed on the smartwatch and changes when the HR level decreases or increases more than one level. After every three participants, we checked whether we had to refine the settings to improve usability, for example accounting for feedback about receiving too many notifications. Ultimately, the sensitivity of the app was set to low (expanding the ranges between levels by multiplying the standard deviation with a 1.5 factor) and the notifying vibrations were given at levels 4 and 5 above baseline. The Sense-IT app also detects (physical) activity categories using the accelerometer and Google activity recognition algorithms, allowing the user to receive notifications for certain activity profiles. In this study, we ended up offering notifications for low activity profiles (i.e., sitting still, walking) only. In the user interface on the smartphone, users can turn the app on and off, and open a timeline of all measurement events and level changes detected by the system. Users can add notifications to events in the timeline and report their subjective level of arousal, which might particularly be useful when tension increases. Users can also define a personalized message that is displayed when their physiological arousal exceeds a predefined level. In this study, this supportive message and an accompanying question to rate subjective stress were displayed at levels 4 and 5. The user interface also presented information about connection and synchronization, as well as a settings page which was protected by a password to prevent unwarranted changes. Screenshots of the Sense-IT app are displayed in Figure 2.

#### 2.4.2. Quantitative measures

##### 2.4.2.1. Pretest-posttest design

###### 2.4.2.1.1. Change measures

At T0, T1 and T2 primary and secondary measures were administered to explore relevant changes on group level. The Aggression Questionnaire-Short Form (AQ-SF; Buss and Perry, 1992), a 12-item 5-point Likert scale self-report questionnaire, was used to assess changes in different types of aggressive behavior over the past 4 weeks: physical aggression, verbal aggression, anger, and hostility. Therapists evaluated aggressive behavior of their patients during the same period with the Modified Overt Aggression Scale (MOAS; Knoedler, 1989), a 4-item observation scale differentiating verbal aggression, aggression against property, auto-aggression, and physical aggression. The therapists based their scores on observed incidents (if applicable), information from others (if applicable), and on patients’ retrospective reports of aggressive incidents during the weekly sessions. Another self-report measure, the Anger Bodily Sensations Questionnaire (ABSQ; Zwets et al., 2014), consisting of 18 items with a 5-point Likert scale, was administered to assess changes in psychophysiological awareness. Furthermore, the Difficulties in Emotion Regulation Scale (DERS; Gratz and Roemer, 2004), a 36-item 5-point Likert scale self-report questionnaire, was administered. This questionnaire is used to assess six dimensions of emotional processing: non-acceptance of emotional responses, difficulty engaging in goal-directed behavior, impulse control difficulties, lack of emotional awareness, limited access to emotion regulation strategies, and lack of emotional clarity.

###### 2.4.2.1.2. Descriptive measures

At T0, several other secondary measures were administered to describe the sample. A self-developed demographic questionnaire was used to gather information regarding age, gender, ethnicity, education, and past offenses. The most recent DSM-5 main psychiatric diagnosis for each participant was retrieved from the electronic patient record. To gain a better understanding of the type and nature of aggressive and antisocial behavior, three other self-report measures were administered at baseline: the Reactive Proactive Questionnaire (RPQ; Raine et al., 2006), Youth Psychopathic Traits Inventory-Short Version (YPI-s; van Baardewijk et al., 2010), and State–Trait Anger Expression Inventory (STAXI-2; Spielberger et al., 1999).

##### 2.4.2.2. Single-case experimental designs

###### 2.4.2.2.1. Self-report measures

During the 4 weeks of the ABA designs, Ecological Momentary Assessment (EMA) was used to assess the emotional state of the participants. For this reason, six questions were designed based on the items of the Positive and Negative Affect Schedule (PANAS; Watson et al., 1988). Participants received prompts to answer these questions twice a day, at predetermined times fitting into the daily schedule of the particular participant. They were asked to rate the extent to which they experienced behavioral control and aggressive behavior (primary measures) as well as anger, aggressive thoughts and physical tension (exploratory measures) during the preceding part of the day. A question investigating feelings of happiness was added to balance the questions. A 5-point Likert scale was used for each question, reaching from ‘very slightly/not at all’ to ‘extremely’.

###### 2.4.2.2.2. Physiological measures

During the 4 weeks of the ABA designs, HR was continuously measured while the Sense-IT app was used. The Sense-IT app also registered the baseline settings, kept track of the levels (i.e., a value between −3 and 5) and the activity profiles (i.e., running, cycling, and sitting still), and whether biocueing was active (phase B) or not (phases A_1_ and A_2_).

#### 2.4.3. Qualitative measures

At T1, a semi-structured interview was conducted. This interview included questions about feasibility and usability of the devices, advantages and disadvantages of the Sense-IT app, and recommendations for further improvement. In this article, we focused on three questions regarding the perceived effectivity of the Sense-IT app on interoceptive awareness, use of coping strategies, and prevention of aggressive incidents. A more in-depth analysis of patients’ perspectives on use and implementation of the Sense-IT app is presented elsewhere (Ter Harmsel et al., 2022a).

### 2.5. Data analysis

#### 2.5.1. Quantitative data analyses

##### 2.5.1.1. Pretest-posttest design

The quantitative data (AQ-SF, MOAS, ABSQ, and DERS) were analyzed using SPSS (version 27, IBM Corp). After checking the normality assumptions for main scales and subscales and given the small sample size (particularly for the comparisons with T2), we decided to use the nonparametric equivalent of the paired *t*-test, the Wilcoxon Matched Pairs Test. To make efficient use of the available data two missing items on the DERS, for two different participants, were replaced by imputing the individual mean score on this questionnaire at that moment of assessment.

##### 2.5.1.2. Single-case experimental designs

In order to analyze the SCED data, all EMA and HR measures were divided into the three phases (A_1_, B, and A_2_), using the track record of the Sense-IT app. For EMA, responses were considered as belonging to the last preceding prompt, unless the response was given less than 30 min before the next prompt. In case of multiple responses within 30 min, the response that deviated the most from the specified prompting time was discarded. In case of phase ambiguities, EMA responses were assigned to the phase to which the majority (>50%) of the period over which they reported (i.e., the time between prompts) belonged. For HR, measurements with specific activity profiles (i.e., running, cycling, car driving) were disregarded from the measurements to focus on HR data in no (i.e., sitting) to limited (i.e., walking) movement scenarios. Furthermore, the HR data was corrected for very low and high values (< 50 bpm and > 190 bpm). To calculate mean and standard deviation per day part, HR data was split into daytime (08:00 AM – 04:59 PM) and evening measures (05:00 PM - 01:59 AM). A day part was considered missing when less than 500 HR measures were present. When participants had no access to the Sense-IT app and its associated devices for at least three days (e.g., due to vacation), the corresponding period was not included in the analysis.

After data preprocessing a visual analysis, considered as the primary method in SCED research (Kazdin, 2019), was performed on the selected EMA variables (i.e., anger, physiological stress, aggressive thoughts, aggressive behavior and behavioral control) and HR variables (i.e., mean and standard deviation). For participants with at least 5 data points per phase (Bolger and Laurenceau, 2013), we graphically compared the direction and rate of change (i.e., the slopes of the regression lines) between the different phases for each variable. We made plots for each participant separately as well as for the entire group of eligible participants. First, we used (R Core Team, 2017) lme function from the nlme package (Pinheiro and Bates, 2000) to apply a multilevel (two-level) piecewise regression approach analyzing the effects between phases per variable, for all the eligible participants on group level. Second, we performed (one-level) piecewise regression analyses per variable for each participant using the R-based MultiSCED web application (Declercq et al., 2020). The unstandardized parameter estimates of each variable at the start of the study (intercept, B0), the developmental effect in the variable over time in a particular phase (time, B1), an immediate variable change when transitioning into the intervention phase (phase, B2) and a comparison of variable change over time in the intervention phase compared to the baseline phase (time x phase, B3) were calculated. For results, we reported the estimates B1 (for both baseline and intervention phase), B2, and B3. To account for Type-I errors, we used a Bonferroni-corrected value of p (*p* ≤ 0.01) by dividing the critical value of p (*α* = 0.05) by the number of comparisons (five). In addition, we assessed effect sizes on group and individual level for the EMA variables for which we expected a specific direction of change (i.e., behavioral control and aggressive behavior) by calculating the Improvement Rate Index (IRD; Parker et al., 2009). We calculated this nonparametric overlapping index, comparing the improvement rate between two phases, using an online single-case effect size calculator (Pustejovsky et al., 2021).

#### 2.5.2. Qualitative data analyses

We organized and analyzed the data of the qualitative interview using Microsoft Excel. Dichotomous responses were described as relative results. Two researchers (JtH and LS) independently ranked the three most informative textual responses regarding interoceptive awareness, use of coping strategies, and prevention of aggressive incidents. The final quotations were selected by discussion between two researchers (JtH and LS), until consensus was reached, and translated from Dutch into English.

## 3. Results

### 3.1. Descriptive characteristics

The majority of the 25 forensic outpatients who participated in this study was male (92%) and born in Netherlands (92%). For most of them, treatment was a mandatory part of their conditional sentence (64%), mainly imposed because of a violent index offense (94%). A large proportion (73%) reported problems in the family of origin: domestic violence and substance abuse were most frequently reported, but criminal behavior and psychological problems were also mentioned. All descriptive characteristics are summarized in Table 2.

**Table 2:** Demographic characteristics (*N* = 25).

Outcome	Mean (SD)	*n* (%)
Age	29.88 (10.51)	
Gender		
*Male*		23 (92%)
*Female*		2 (8%)
Migration background		
*First-generation migration background*		2 (8%)
*Second-generation migration background*		14 (56%)
*Dutch background*		9 (36%)
Educational background		
*None*		1 (4%)
*Primary education*		4 (16%)
*Junior secondary education*		14 (56%)
*Senior secondary education*		6 (24%)
Indication of mild intellectual disability (SCIL)		9 (36%)
Main psychiatric classification according to DSM-5		
*Disruptive disorder*		10 (40%)
*Substance use disorder*		2 (8%)
*Posttraumatic stress disorder*		2 (8%)
*Borderline personality disorder*		2 (8%)
*Other specified personality disorder*		7 (28%)
*Other disorder*		2 (8%)
Mandatory treatment		16 (64%)
Past offenses (official records)		
*0*		8 (32%)
*1 or 2*		6 (24%)
*3 to 5*		4 (16%)
*6 to 10*		3 (12%)
*More than 10*		4 (16%)
Aggression type (RPQ)		
*Reactive aggression*	14.72 (4.39)	
*Proactive aggression*	7.53 (4.61)	
Anger and anger regulation (STAXI-2)		
*State anger*	18.80 (6.85)	
*Trait anger*	23.76 (6.97)	
*Anger expression index*	50.72 (10.81)	
Psychopathy (YPI-s)		
*Interpersonal dimension*	12.40 (4.73)	
*Affective dimension*	11.48 (4.39)	
*Behavioral dimension*	15.56 (3.35)	

### 3.2. Pretest-posttest results

First, we analyzed the results of the quasi-experimental designs with pre-, post-, and follow-up measurements. The mean scores and standard deviations on clinical outcomes aggression (AQ-SF and MOAS), insight in anger bodily sensations (ABSQ), and emotion regulation difficulties (DERS), for each moment of assessment, are presented in Table 3. For statistical testing, data of 20 participants could be used to explore the difference between T0 and T1; and data of 14 participants for the differences between T0 and T2, and T1 and T2. The results of Wilcoxon Matched Pairs tests indicated that self-reported aggression decreased significantly between T0 (Median = 35.5) and T1 (Median = 31.5); *Z* = −2.043, *p* = 0.041. No significant decreases in aggression were found between the other moments of assessment. For therapist reported aggression level, emotion regulation difficulties, and insight in anger bodily sensations no significant differences were found between pre-, post- and follow-up assessment. For this sample, three of the outcome measures changed in the expected direction between T0 and T1, and one measure (anger bodily sensations) changed in the opposite direction.

**Table 3:** Overview of clinical outcomes at pre-, post- and follow-up measurement.

Outcome	T0 (*N* = 25) Mean (SD)	T1 (*N* = 20) Mean (SD)	T2 (*N* = 14) Mean (SD)
Aggression, self-report (AQ-SF)	32.44 (9.51)	30.80 (8.68)	28.07 (10.40)
Aggression, therapist-report (MOAS)	5.24 (5.46)	3.48 (3.30)	4.92 (6.46)
Emotion regulation difficulties (DERS)	100.40 (25.55)	93.14 (25.52)	89.20 (24.98)
Anger bodily sensations (ABSQ)	49.96 (16.12)	45.70 (13.74)	42.07 (14.11)

### 3.3. Single-case experimental design results

Next, we analyzed the results of the ABA designs. In order to select the participants with sufficient data points, we started by investigating data availability. One participant did not start using the Sense-IT app, one participant quit after phase A_1_ and seven participants stopped using the app after phase B. The compliance to EMA, defined as the ratio of the number of completed EMA questions in relation to the total number of EMA prompts per phase, ranged from 43.7% in Phase A_1_, to 24.7% in Phase B and 16.0% in Phase A_2_. For EMA, only 3 participants met the criterion of at least 5 data points for all phases. The compliance to HR measurement, the ratio of available daytime or evening measures in relation to the maximum amount of these measures per phase, ranged from 38.5% in Phase A_1_, to 29.9% in Phase B and 13.5% in Phase A_2_. For HR, none of the participants had sufficient measurements in all phases. Therefore, only the results of the baseline phase (A_1_) and intervention phase (B) were used for further analysis: for EMA, 9 participants had sufficient data in phase A_1_ and B; for HR this applied to 10 participants.

**Figure 3:**
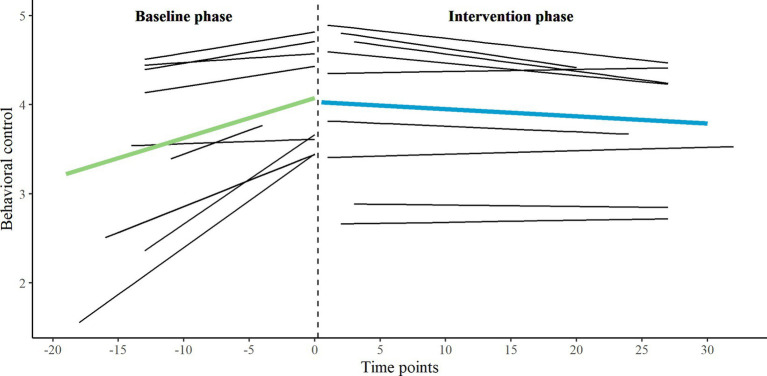
Combination of one- and two-level regression results for primary EMA measure behavioral control, measured twice a day on a 5-point Likert scale, in baseline phase A_1_ and intervention phase B.

**Figure 4:**
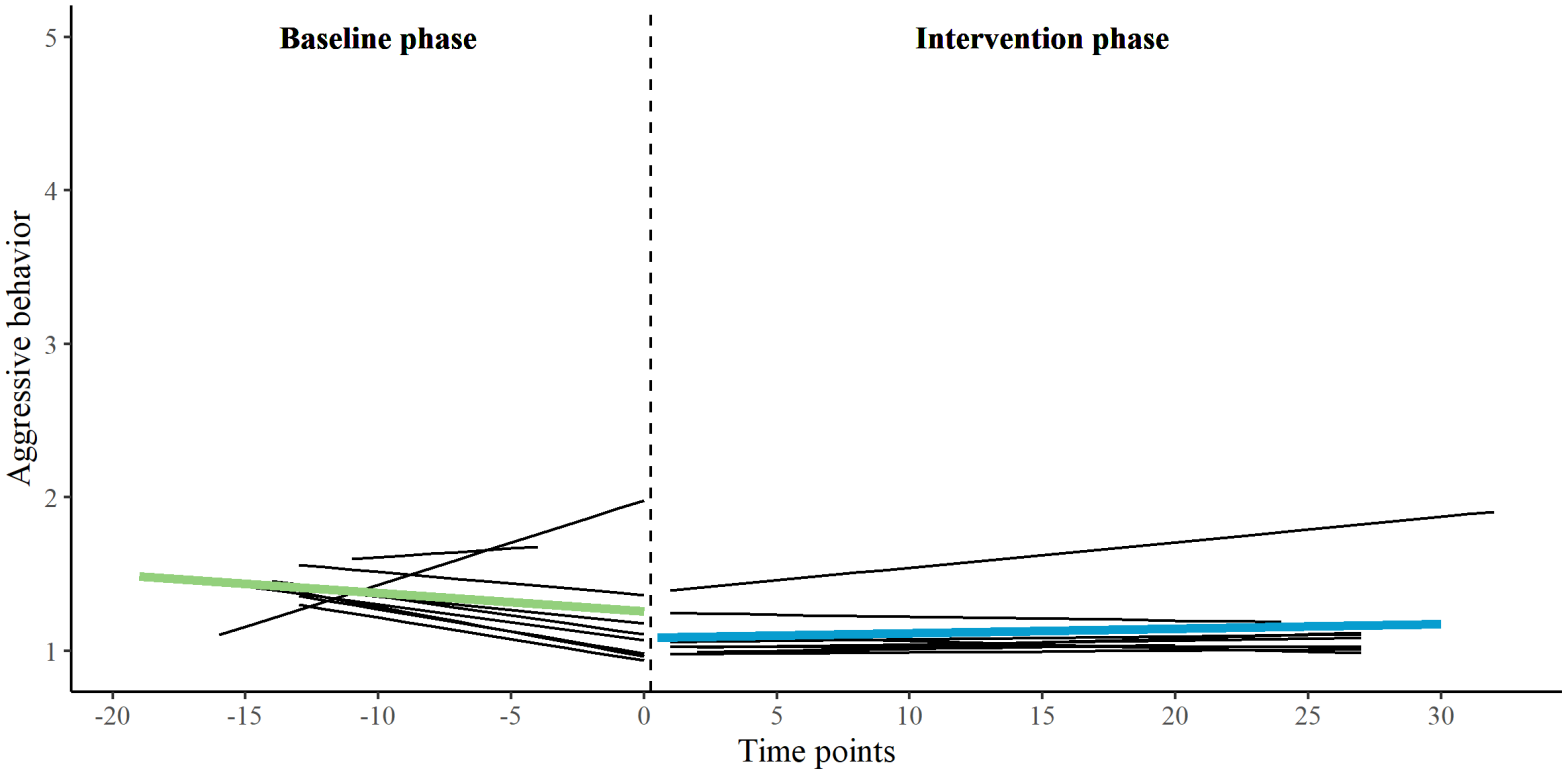
Combination of one- and two-level regression results for primary EMA measure aggressive behavior, measured twice a day on a 5-point Likert scale, in baseline phase A_1_ and intervention phase B.

First, we applied a multilevel piecewise regression approach (two-level) and visual analyses to analyze the effects between phases for five EMA variables and two HR variables on group level, using the data of all eligible participants. We inspected B1 (for both baseline and intervention phase), B2 and B3. On group level, we found no significant developmental effects (neither for baseline nor for intervention phase), no significant immediate changes when transitioning into the intervention phase, and no significant interaction effects on any of the variables. All group-level parameter estimates are presented in the Supplementary material. The individual and group-level results of the two primary EMA measures, behavioral control, and aggressive behavior, are illustrated in Figures 3, 4. For exploratory measures, see Supplementary material. Improvement rate differences (IRDs) for these outcomes on group level were.29 for behavioral control (increasing direction) and 0.26 for aggressive behavior (decreasing direction), indicating a small effect size of the combination of biocueing intervention and ART on these outcome measures (Parker et al., 2009).

Subsequently, piecewise regression analyses and visual analyses were conducted for each of the eligible participants separately (one-level), using MultiSCED. Significant developmental effects (for both baseline and intervention phase), immediate changes when transitioning into the intervention phase, and interaction effects are reported in the Supplementary material. An overview of all unstandardized parameter estimates for each participant is available upon request from the first author.

Improvement rate differences (IRDs) for the two primary EMA outcomes on the individual level ranged from 0.05 to 0.51 for aggressive behavior (decreasing direction) and from.05 to.55 for behavioral control (increasing direction), indicating small effect sizes with some exceptions to moderate (Parker et al., 2009).

Finally, we zoomed in on the three participants in which we found visually interesting patterns and significant interaction effects to enhance clinical understanding of the results. The names of the participants are fictitious.

#### Ryan

**Figure 5:**
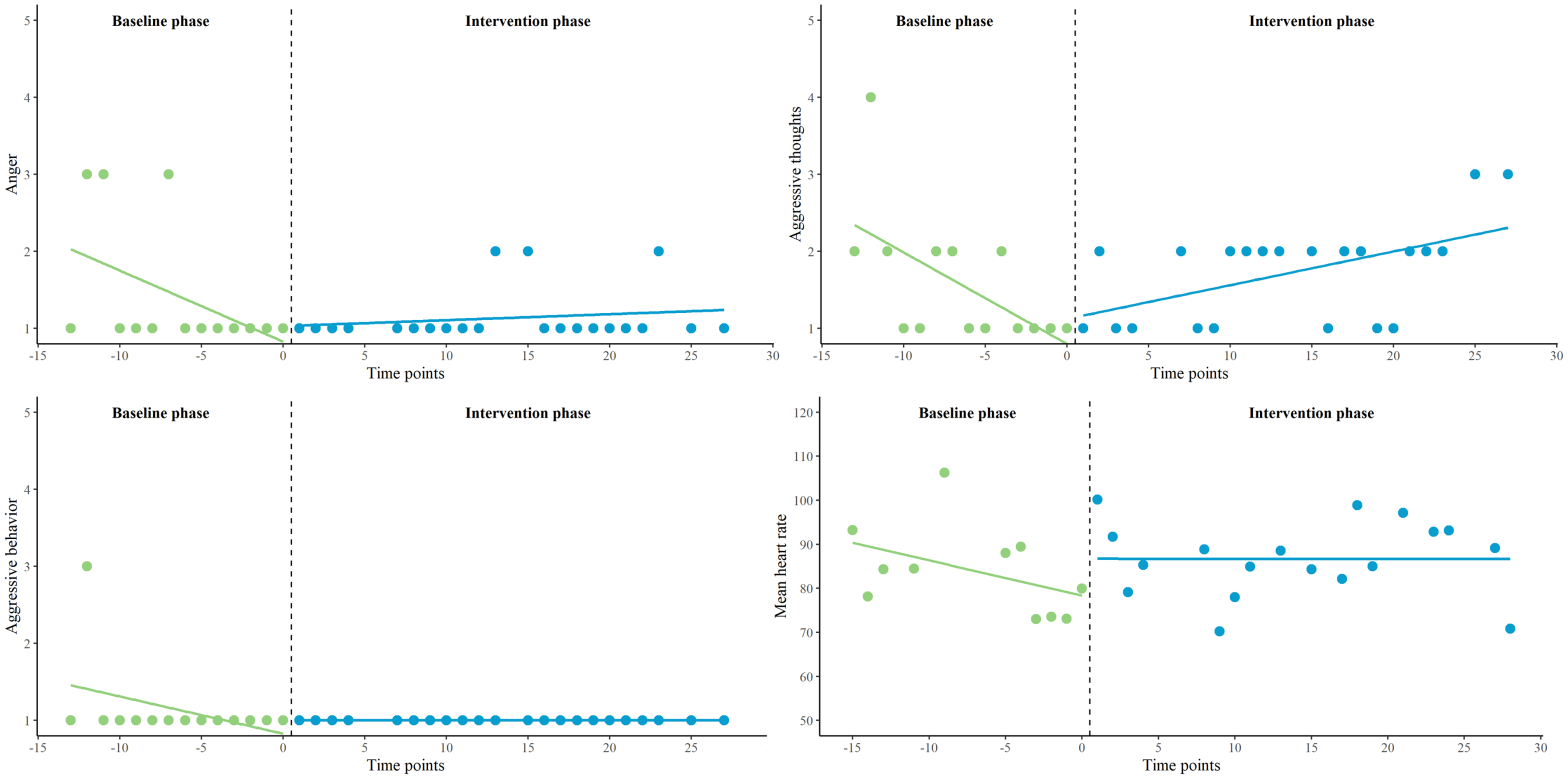
Representation of Ryan’s EMA measures (anger, aggressive thoughts and aggressive behavior) and HR (average), measured twice a day on a 5-point Likert scale, in baseline (A_1_) and intervention phase (B).

This participant, aged between 30 and 35, had severe aggression regulation problems. At baseline (T0), Ryan achieved a high score (9th decile) on the AQ-SF compared to other outpatients with violent behavior. He reported predominantly reactive aggression on the RPQ. Furthermore, Ryan experienced many emotion regulation difficulties (DERS). His anger expression index on the STAXI-2 (95th percentile) shows that he tended to express his emotions more outward than inward, and that his ability to regulate his emotions was very low. Using the piecewise regression results and visual analysis (see Figure 5), his feelings of anger, aggressive thoughts, aggressive behavior and mean HR all seem to decrease during the baseline phase (A_1_). Most remarkable in the intervention phase (B) is the increase in aggressive thoughts. Both the decrease in aggressive thoughts during the baseline phase and the increase in the intervention phase reached statistical significance. However, Ryan reported that he did not express these thoughts in aggressive behavior, as indicated by the flat line. When the patterns in both phases were compared, his outcomes regarding aggressive thoughts were significantly in favor of the baseline phase. No significant differences between phases were found on other variables. At post-test (T1) Ryan reported a substantially lower score on the AQ-SF (6th decile) compared to baseline, but a higher score on the DERS. He reported that the Sense-IT biocueing app did not work for him. He noticed no effect of biocueing on his awareness of physiological signals of tension, use of adequate coping, or prevention of aggressive behavior. Ryan mentioned that the Sense-IT app signaled tension when there was none and did not signal tension when there was; questioning the accuracy of the feedback.

#### Eric

**Figure 6:**
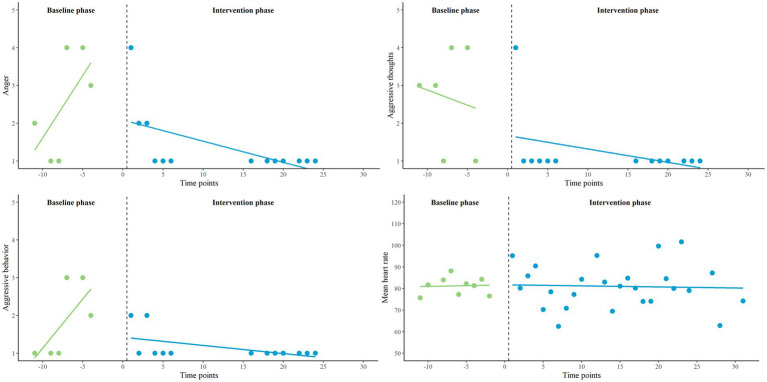
Representation of Eric’s EMA measures (anger, aggressive thoughts and aggressive behavior) and HR (average), measured twice a day on a 5-point Likert scale, in baseline (A_1_) and intervention phase (B).

This participant, aged between 40 and 45, also struggled with aggression regulation problems. At baseline, he scored average on the AQ-SF compared to the norm group (6th decile). Eric also reported predominantly reactive aggression on the RPQ. He experienced emotion regulation difficulties (DERS) to an average degree. The anger expression index (81st percentile) indicates that Eric also tended to direct his anger more outward than inside, and that his regulation skills were quite low. The piecewise regression results and visual analysis (see Figure 6) illustrate that anger and aggressive behavior seem to increase in the baseline phase (A_1_), while aggressive thoughts seem to decrease. The increase in aggressive behavior reached statistical significance. In the intervention phase (B), these variables all seem to change in decreasing direction. None of these decreases reached statistical significance. Mean HR seems stable. When the patterns in aggressive behavior were compared between both phases, his outcomes did significantly favor the intervention phase. No significant differences between phases were found on other variables. Compared to baseline, Eric also achieved a lower score on the AQ-SF (4th decile) at post-test. His score on the DERS remained the same. Eric reported no effect of biocueing on his awareness of physiological signals of tension, use of adequate coping, or prevention of aggressive behavior. He stated that he was not inclined to act upon the physiological feedback he received.

#### Joshua

**Figure 7:**
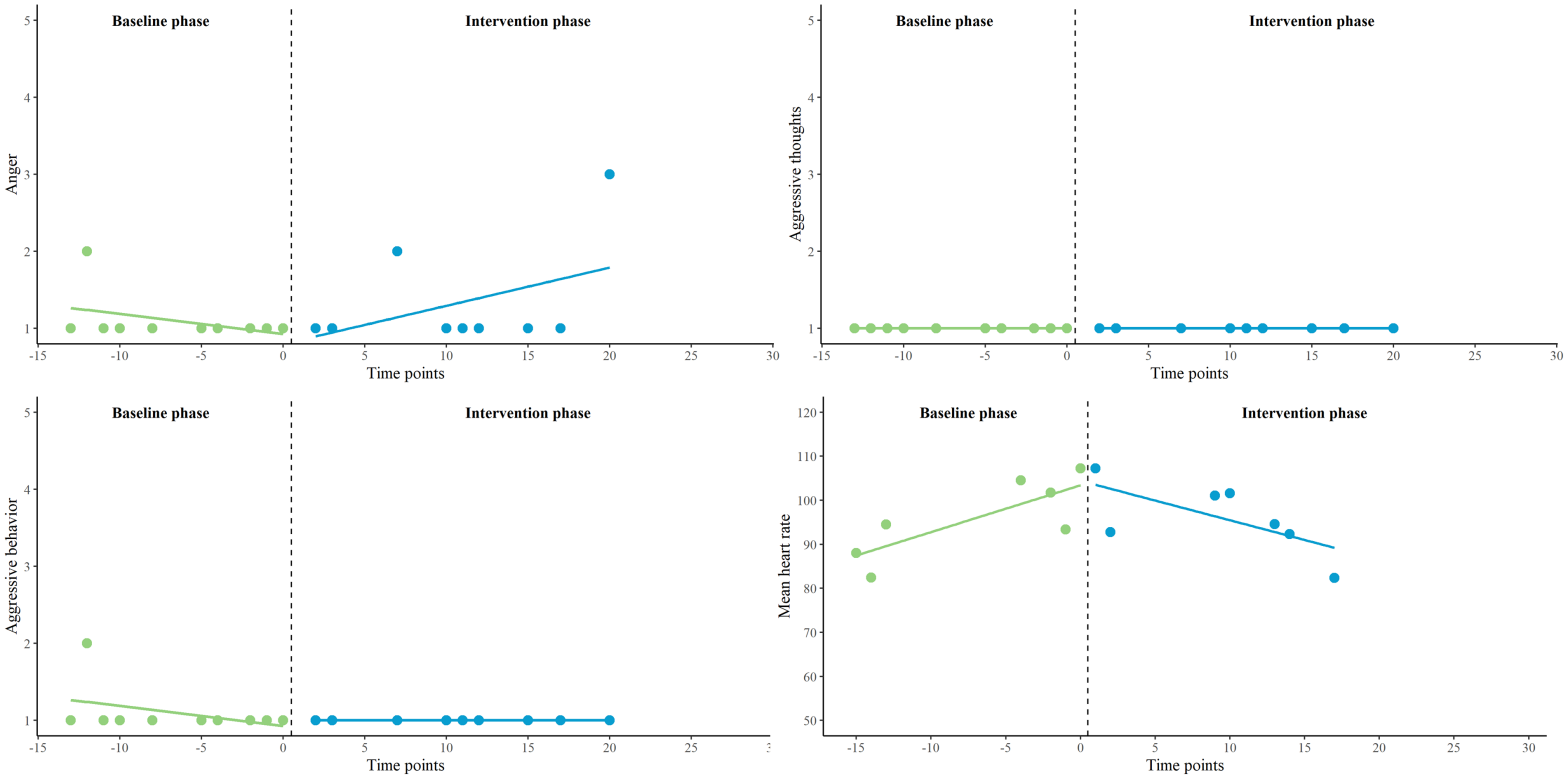
Representation of Joshua’s EMA measures (anger, aggressive thoughts and aggressive behavior) and HR (average), measured twice a day on a 5-point Likert scale, in baseline (A_1_) and intervention phase (B).

This participant, aged between 20 and 25, also had aggression regulation problems. At baseline, Joshua scored average compared to other outpatients with violent behavior (6th decile). He reported similar levels of reactive and proactive aggression on the RPQ. Joshua experienced emotion regulation problems (DERS) to an average degree. His anger expression index (60th percentile) indicated that he expressed his anger in both directions, and more inward compared to the other Ryan and Eric. His regulation skills were average to good. The piecewise regression results and visual analysis (see Figure 7) revealed that he had experienced little anger, aggressive thoughts, and aggressive behavior. No significant in- or decreases in phases and no significantly different patterns between phases were found for these variables. However, the decrease in mean HR in the intervention phase (B) significantly differed from the increase in the baseline phase (A_1_), which might favor the intervention phase. Compared to baseline, Joshua scored slightly lower on the AQ-SF (5th decile) at post-test. The score on the DERS also decreased slightly, but was still in the same range. Joshua did notice a positive effect of biocueing on his awareness of physiological signals of tension. He explained that the app helped him not to get stuck in anger by using adequate coping strategies, such as seeking distraction and clearing his mind. He did not notice an effect on prevention of aggressive behavior as he felt he had not been aggressive during the research period.

### 3.4. Qualitative results

**Figure 8:**
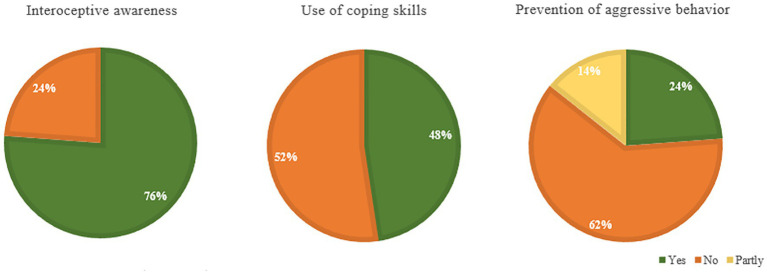
Perceived effectivity of the Sense-IT app on interoceptive awareness, use of coping skills and prevention of aggressive behavior according to forensic outpatients.

Qualitatively, we focused on the perspectives of patients regarding the perceived effectivity of the Sense-IT app on interoceptive awareness, use of coping strategies, and prevention of aggressive behavior. The responses of the participants can be found in Figure 8. As shown, the majority of the forensic outpatients (76%) reported increased insight into physiological signals of tension, after using the app. For example, one of these participants mentioned: *“I felt the tension building and when it [the watch] started to vibrate I recognized that I was angry and went to do something else”* (P16) and another reported: *“That my heart rate is often high even though I do not think so myself*.*”* (P3) However, other patients indicated: *“I was often alerted to tension when I already knew it*.*”* (P21) and “*The app sends false notifications: it vibrates while nothing is going on and not when you are stressed out”* (P1). Approximately half of the participants (48%) felt supported by the Sense-IT app to use coping strategies in order to reduce stress. For example, one participant reported: *“When the watch vibrated I took the notification into account*, *for example by coming to myself and taking a breath*.*”* (P17) and another said: *“I went to do something else and cleared my mind instead of dwelling in anger*.*”* (P10). In some cases, the coping strategies used may have shed light on less adequate behavioral patterns:*“[When the watch vibrated] I went to smoke a joint and got calmer*. *I also went gaming*.*”* (P9). Another participant mentioned: *“I will not be guided by a watch*. *Did look at the notification*, *but did not react to it*. *Screw it*, *I’ll just stay angry*.*”*(P8), indicating that at least some motivation to change behavior and openness to feedback are necessary prerequisites to benefit from the app. Moreover, it shows that it can be difficult for some patients to distinguish anger from aggression. Finally, about one-third of the participants (38%) reported that using the app might have helped them to prevent aggressive behavior in some cases. One participant responded: “*Maybe*. *By participating in this study*, *I became more aware to reduce my anger when the watch indicated*, *for example when stressed at work*.*”* (P25). Other participants mentioned that they were able to calm themselves in some cases, but not in all: *“Sometimes I took it easy*, *but sometimes I was just angry and did not do anything*.*”* (P9) or: *“Sometimes you do not think about the watch*, *then things go fast and something happens*.” (P7). Another participant stressed the boundaries of an app: *“It takes more than that*. *When I am very aggressive*, *I will not be stopped by a vibrating watch or a mobile with questions”* (P16). Furthermore, several participants reported increased stress and irritation by the number of notifications and the perceived inaccuracy of the app: *“Actually*, *it increased my frustration and irritation*. *I had to actively suppress not throwing the thing away*.*”*(P21). Other participants indicated that this last question was difficult to answer as they had not experienced aggressive outbursts in the past period.

## 4. Discussion

In the current study, we explored the effects of a new version of the Sense-IT biocueing app on interoceptive awareness, emotion regulation, and aggressive behavior in a forensic outpatient population. In this study, the Sense-IT app was added to regular ART. Quantitatively, we examined group changes on measures of aggression, emotion regulation and insight in anger bodily sensations between pretest, posttest, and follow-up (pretest-posttest design), as well as group and individual changes in behavioral control, aggressive behavior, anger, aggressive thoughts, physiological tension and HR in the intervention phase compared to the baseline phase (SCEDs). In addition, we qualitatively assessed patient-reported changes in interoceptive awareness, use of coping strategies, and prevention of aggressive incidents associated with the use of the Sense-IT app.

The results of the pretest-posttest design showed a significant decrease in self-reported aggression between pretest and posttest, indicating a positive effect associated with the combination of the Sense-IT biocueing intervention and ART. In addition, although on visual inspection emotion regulation difficulties decreased in this sample, no significant effects were found. Furthermore, no significant differences were found for therapist-reported aggression level and insight into anger bodily sensations. The quasi-experimental nature of this design prohibits attribution of the found effect to either the combined intervention or the regular therapy alone. However, the fact that the significant decrease in aggression was only found between pretest and posttest (the period the biocueing was added) and not for the comparison with follow-up (the period regular therapy was continued without the biocueing intervention), is interesting, and could be further investigated in future studies using a controlled research design.

Due to low compliance to EMA and a low amount of sufficient HR measures per day part, data analysis of the SCEDs was limited to EMA data of 9 participants and HR data of 10 participants. Focusing on the primary measures, aggressive behavior and behavioral control, an interaction effect favoring the biocueing intervention was found for one participant (a decrease in aggressive behavior in the intervention phase compared to an increase in the baseline phase). Regarding exploratory measures, a reverse pattern was found in another participant (an increase in aggressive thoughts in the intervention phase compared to a decrease in the baseline phase). For another participant, mean HR decreased in the intervention phase compared to an increase in the baseline phase. On group level, we found no significant developmental effects in the baseline and intervention phase, no significant immediate changes when transitioning into the intervention phase, and no significant interaction effects on any of the variables. Overall, effect sizes were small, with some individual exceptions to moderate.

In contrast, the qualitative results do indicate positive changes related to the use of the Sense-IT biocueing app, such as increased interoceptive awareness among the majority of the participants, perceived support in the use of coping strategies by half of the participants, and prevention of some aggressive incidents by one-third. Although most participants reported increased insight into physiological signals of tension, results show that the step from insight toward adequate emotion regulation requires more attention. Furthermore, results seem to indicate that a certain amount of motivation to change behavior and openness to feedback are necessary to benefit from the just-in-time behavioral support delivered by the Sense-IT app. Although the usability of the Sense-IT app was acceptable (Ter Harmsel et al., 2022a), some patients reported increased stress and irritation by the number of notifications and the perceived inaccuracy of the app.

In sum, out of the subgroup of patients who qualitatively reported positive changes associated with the use of the biocueing intervention, we only found a significant quantitative change favoring the intervention phase for one patient with sufficient data for single-case analysis. We therefore conclude that, whereas the quantitative results of the pretest-posttest design and the qualitative results indicated positive changes associated with the combination of biocueing intervention and ART, the repeated ambulatory measurements of the SCEDs do not indicate a clear effect favoring the addition of a biocueing intervention.

First of all, we would like to shed some light on the findings related to interoceptive awareness. The awareness of bodily sensations has been identified as an important component in the process of emotional awareness (Lane and Schwartz, 1987; Calì et al., 2015), which is, in turn, an essential building block for adequate emotion regulation (Füstös et al., 2013; Gross and Jazaieri, 2014). In our study, the majority of patients qualitatively reported increased interoceptive awareness, although quantitively no significant difference and insight in anger bodily sensations was found. Several factors might have contributed to this finding. First, patients might have developed a more accurate view of their physiological sensations in angry interactions by using the biocueing intervention. Second, while interoceptive awareness primarily concerns perception, anger- related interoceptive awareness also entails some interpretation of bodily signals (Bellemans et al., 2018), and can therefore be considered a more demanding skill. Third, some patients had difficulty differentiating between components of the Sense-IT biocueing app (delivering a real-time visualization of their heart rate level and just-in-time behavioral support) and the integrated daily EMA questions that were used for research purposes. Since some patients reported that ‘the questions’ (unspecified) were really helpful to reflect on their emotional state, the qualitative measure of interoceptive awareness associated with the use of the biocueing intervention may have been somewhat diluted by perceived increases in emotional awareness by responding to the EMA questions across all phases. Fourth, recent research states that (Garfinkel et al., 2015; Forkmann et al., 2016) interoceptive awareness should be considered as a metacognitive process, integrating both interoceptive sensibility (i.e., self-evaluated assessment of subjective interoception) and interoceptive accuracy (i.e., performance on objective tests of heartbeat detection). According to this model of interoception, our study focused on the facet of interoceptive sensibility and thereby lacked information regarding interoceptive accuracy and interoceptive awareness as a metacognitive process. Since the feedback was perceived as inaccurate by a substantial part of the patients, which may be partly related to technical issues but may also be explained by limited interoceptive capabilities, understanding the role of interoception in using biocueing among forensic outpatients could have been enhanced if we had included an interoceptive accuracy measure and had used a clearer definition of interoceptive terms (Khoury et al., 2018).

Second, we would like to focus on the findings regarding emotion regulation. Although half of the participants qualitatively reported that they felt supported by the app to use coping strategies in order to reduce stress, and on visual inspection emotion regulation difficulties decreased in this sample, no significant effects associated with the combination of biocueing intervention and ART were found in the pretest-posttest design. We also found no group or individual increases in behavioral control favoring the biocueing intervention in the SCEDs. Several factors might have contributed to these findings. First, motivation to change and feedback receptivity varied among the participants. Although these factors might not be required to take advantage of the component of the Sense-IT app aimed at increasing interoceptive awareness, feedback receptivity turned out to be quite essential to benefit from the behavioral support component of the Sense-IT app, even among non-psychiatric samples without motivational difficulties (Lentferink et al., 2021). For future use, it is therefore important to assess whether patients are open to receiving feedback and willing to try out different emotion regulation strategies. Related to this, some patients had (very) high expectations of what the app should deliver, which might have led to disappointment when subjectively experienced tension was not noticed or when they received behavioral support messages while they felt relaxed and did not notice tension. Although biocueing interventions can identify substantial increases in arousal by measuring HR, they are unable to provide a flawless recognition of subjectively experienced stress and cannot determine valence in order to specify emotion categories (Siegel et al., 2018). As suggested in recent research in which patients’ reported similar feedback (Bosch et al., 2022), a more detailed explanation of biocueing might help to let patients realize that additional appraisal has to be exerted by themselves. Since some patients reported less adaptive coping behaviors in response to behavioral support messages, discussing and drafting the personalized message should be integrated into therapy and given more attention. Furthermore, some patients might benefit more from integrated relaxation exercises or gamified interventions instead of a text message (Bakker et al., 2016). This all emphasizes the need to integrate new interventions in regular treatment and to tailor these interventions to patient-specific needs (Kip et al., 2018).

Third, we would like to reflect on the findings regarding aggressive behavior. First of all, as patients reported in the qualitative part of the study, it is hard to determine if (hypothetical) aggressive incidents had been prevented and if so, whether that could be associated with the use of the biocueing intervention. Regarding aggression, the pretest-posttest results did indicate a significant decrease in aggression associated with the combination of biocueing intervention and ART. A significant decline in aggressive behavior favoring the biocueing intervention was found in one participant in the SCEDs However, no group effects were found using the repeated ambulatory measurements of the SCEDs, and findings were not supported by therapist-reported aggression level. Several factors are worth noting. First, the EMA responses showed a low prevalence of aggressive behavior in most patients. This created a bottom effect in some patients. Whether this is related to social desirability, lack of concept clarity, or an actual low incidence rate remains unclear. Second, the added value of therapist-reported aggression levels should be considered limited as these scores were not only based on actually observed aggressive incidents but also on patients’ reports thereof during the weekly therapy sessions. As some social desirability might have been at play in both therapist- and patient-reported aggression (Barry et al., 2017), patients’ reports may have rendered even more accurate information on aggressive behavior in this study given the perceived anonymity of these reports (Lobbestael, 2015). All these factors emphasize the challenges of aggression assessment among forensic outpatients, especially in outpatient settings (Lobbestael, 2015). Furthermore, some patients seemed to mix up anger and aggressive behavior, as if their treatment goal was never to experience anger again. This again stresses the need for integration of the app into therapy and the importance of psycho-education, problematizing aggressive behavior but normalizing feelings of anger. Finally, as the findings of the case studies provide insufficient support for the idea that biocueing interventions might be particularly beneficial for patients with predominant reactive aggression, this topic needs further investigation.

### 4.1. Strengths and limitations

Several strengths and limitations could be applied to this study. A noteworthy strength is that this study is one of the first in which a biocueing intervention, using psychophysiological measures, is used as an addition to regular treatment in a complex forensic outpatient sample with anger regulation difficulties. As main end-users, the forensic outpatients were involved throughout the developmental process, delivering us with valuable feedback and recommendations for future use of the app. Another strength is the use of a mixed-methods design. Integrating quantitative and qualitative data, on group and individual level, enabled us to explore the effects of the combination of a biocueing intervention and ART and to extract relevant information for further development and implementation in clinical practice.

Our study also had several limitations. First of all, we did not use a control group. Although we did use control and experimental phases in the SCEDs, the lack of a control group prohibits attribution of the found pretest-posttest effects to either the combined intervention or the regular therapy alone. The second limitation is related to compliance. For example, not all patients started with the biocueing intervention and several participants had difficulty answering the EMA questions twice a day. Since we expected these challenges, given the characteristics of forensic outpatient populations, we tried to enhance compliance by sending reminders and contingency management (doubling the amount on the gift card when more than 75% of the EMA questions was answered), as suggested in experience sampling literature (Myin-Germeys and Kuppens, 2021). Despite these efforts, compliance to the intervention and the SCEDs remained low, particularly in the follow-up phase (A_2_). Therefore, only the results of the baseline phase (A_1_) and intervention phase (B) could be analyzed, for a select group of patients. Fortunately, most patients, including those who prematurely stopped using the app, still participated in the post-measurements of the pretest-posttest design. Although we thereby reduced the risk of bias in the quantitative and qualitative group results, some patients with negative experiences did still just return their devices and reported that they did not want to participate anymore. Another limitation related to the SCEDs is the fact that we were unable to (randomly) assign participants to different lengths of the baseline phase. Since we had already difficulty engaging participants, we had to let go of specific days that would match the research design but would not fit in the schedule of the participants. Since we only found small effects the impact of the missing multiple baseline analysis seems negligible. Fourth, limitations in the usability of the Sense-IT app may have overshadowed the effects of this additive intervention. Connectivity issues, other design preferences, restricted ability to customize the settings, use of a research-owned smartphone, and limited battery life of the smartwatch are disadvantages that are extensively discussed in another study (Ter Harmsel et al., 2022a). For now, we highlight the fact that participants kept reporting that they received too many notifications, even after we adapted the sensitivity, the levels at which notifying vibrations were given, and the activity categories in which notifications were provided. Some participants reported that they received many notifications when they engaged in only minor physical activities. Others reported that they received many notifications in intense physical activities (e.g., sports, intensive work), indicating that these were not recognized by the activity recognition algorithms. Furthermore, displayed activity profiles not always corresponded with their actual activity. In some patients, these notifications increased stress, led to frustration, and may have resulted in early termination of the research project. Important to note is that, outside of a strict research setting, patients would be able to adapt the sensitivity, levels, and activity profiles themselves, as well as to use the app on their own smartphone, which is expected to increase usability. Furthermore, the presentation of activity profiles, as recognized by the smartwatch using Google algorithms, has been modified in a new version of the Sense-IT app. As the number of notifications is directly related to the standard deviation of the baseline measurement, further refinement (e.g., a longer measurement with increased heart rate variety) of the here introduced baseline measurement procedure should be part of future biocueing studies. Moreover, although wrist based PPG measurements generally produce accurate heart rate estimations in various age groups (Chow and Yang, 2020), it would be advisable for future studies to have access to independent validation of available wearables on the basis of standardized validation protocols (e.g., Van Lier et al., 2019). Fifth, both the therapist-reported aggression levels and patients’ self-reported aggressive behavior might have been prone to memory and social desirability biases, which stresses the difficulty of assessing aggressive behavior in outpatient settings. Sixth, the use of EMA questions may not only have had an impact on the qualitative measure of interoceptive awareness, but might also have impacted the entire ABA design. More specifically, the fact that forensic outpatients who often have difficulty reflecting on their emotions and behaviors were facilitated by the daily questions to do so, may have increased awareness of emotions, which may have had therapeutic effects as well. The interpretation of the effects of the biocueing intervention may therefore have been complicated by the use of experienced sampling in this research design. Seventh, although patients and therapists were involved in the entire developmental process of the Sense-IT app, the app was not an integral part of therapy in this study. This limited integration in therapy may have had a negative impact on the results.

### 4.2. Implications for research and clinical practice

Since it is known that a lot of end-users stop using a mental health app if their goals and preferences are not met (Torous et al., 2019), it is important to create more realistic expectations by providing patients with a more detailed explanation of biocueing as well as to improve the usability of the Sense-IT app. A substantial amount of recommendations have yet already been implemented in a new version of the Sense-IT app. Further refinement of the baseline measurement procedure is an important and necessary step, both to increase usability and to facilitate therapists and patients. Some other usability issues, e.g., the limited battery life of the smartwatches and imperfections in activity recognition, might get solved by technological advances in the future. For future research, it would be relevant to further investigate which patients benefit from a biocueing intervention that is integrated in therapy. Important characteristics to be considered are for instance aggression type, feedback receptivity, and mandatory or voluntary treatment. In addition, it should be assessed when and for how long the intervention should preferably be used. These research directions are in line with the shift toward developing and delivering personalized interventions precisely at moments of need (Bidargaddi et al., 2020). Collaboration between research groups and implementation of multicenter trials are encouraged to increase the sample size. In forensic populations, where demanding traditional research methods are often not feasible, SCEDs should be considered. Given our experiences, we recommended selecting measures that are less sensitive to floor or ceiling effects. When EMA is used, our advice would be to clearly distinguish the research component from the studied intervention. Furthermore, to gain a deeper understanding of the role of interoception in biocueing, we suggest using a combination of measures related to different facets of this concept. Finally, we cannot stress enough the importance of integration of the intervention in therapy. In line with the feedback of the forensic outpatients indicating a need for more personalized use (i.e., on their own smartphone, only in specific circumstances, for longer or shorter periods, with the ability to customize the settings themselves), we encourage therapists and patients to use and evaluate the Sense-IT biocueing app in everyday practice.

### 4.3. Conclusion

In sum, the qualitative results indicate that the use of a biocueing intervention as an addition to regular ART could be considered a helpful means to increase interoceptive awareness among forensic outpatients. Furthermore, during the combination of this new intervention and regular ART significant decreases in self-reported aggressive behavior were observed. However, results of the repeated ambulatory measurements of the SCEDs do not indicate a clear effect favoring the addition of a biocueing intervention. On group level, no significant effects were found. On the individual level, effects favoring the intervention condition were only found for two participants. Decreasing compliance to the demanding research design, the possible therapeutic effects of the daily EMA questions, and limitations in both usability and integration in therapy, might have impacted the results and hampered interpretability. Future research and development should focus on increasing usability, tailoring the intervention to individual needs, and on integration into therapy. Furthermore, research should further investigate the individual characteristics (i.e., aggression type, feedback receptivity) associated with effective support by the Sense-IT app, as the use of personalized treatment interventions in clinical practice, including new technological interventions, is only expected to increase in the coming years.

## Data availability statement

The datasets presented in this article are not readily available because of participants privacy. Requests to access the datasets should be directed to the corresponding author.

## Ethics statement

The studies involving human participants were reviewed and approved by METC, VUmc: NL63911.029.17. The patients/participants provided their written informed consent to participate in this study.

## Author contributions

JH, MN, AP, and TP conceived the entire study and developed the study designs. JH and LS performed the qualitative analysis. JH coordinated the data collection and processing, wrote the manuscript, and analyzed the quantitative data in close cooperation with MN, who provided an expert assessment and contributed R code. MN, LS, TP, AG, and AP critically revised the manuscript. All authors read and approved the final manuscript.

## Funding

This study was performed within a larger research project, financially funded by the Dutch Ministry of Justice and Security (no grant code available).

## Conflict of interest

The authors declare that the research was conducted in the absence of any commercial or financial relationships that could be construed as a potential conflict of interest.

## Publisher’s note

All claims expressed in this article are solely those of the authors and do not necessarily represent those of their affiliated organizations, or those of the publisher, the editors and the reviewers. Any product that may be evaluated in this article, or claim that may be made by its manufacturer, is not guaranteed or endorsed by the publisher.
