# Supplementary material for: Exploring the effects of a wearable biocueing app (Sense-IT) as an addition to aggression regulation therapy in forensic psychiatric outpatients
Source: Front Psychol. 2023 Mar 10;14:983286. doi: 10.3389/fpsyg.2023.983286 (PMC10036768; doi:10.3389/fpsyg.2023.983286)
Supplement: Supplementary file 1 [file Data_Sheet_1.pdf]

Supplementary Material

Supplementary Table 1. Establishment of the baseline procedure.

**Group 1 (adults)**	
Baseline procedure:<br>- Started at end of baseline assessment (T0), in everyday life.<br><br>Settings:<br>- Sensitivity: normal<br>- Notifications at: level 3,4,5<br>- Notifications during: all activity profiles, except car driving	"The mean heart rate should preferably be around 75; and SD should preferably be higher than 7. If heart rate is very different, consider a new baseline measurement. If SD is remarkably low (e.g., < 3), consider adjustment. Never forget to record adjustments."

**Group 2 (adults)**	
Baseline procedure:<br>- During baseline assessment (T0), with 1 min. of walking activity<br><br>Settings:<br>- Sensitivity: low<br>- Notifications at: level 4,5<br>- Notifications during: all activity profiles, except car driving	"The mean heart rate is preferably around 85; the SD should preferably be higher than 7.5. If the heart rate is higher than 100, record the results and then start a new baseline measurement. If the SD is notably low (< 3) or high (> 20), again note the outcomes and then start a new baseline measurement."

**Group 3 (young adults)**	
Baseline procedure:<br>- During baseline assessment (T0), with 1 min. of walking activity<br><br>Settings:<br>- Sensitivity: normal<br>- Notifications at: level 4,5<br>- Notifications during: all activity profiles, except car driving	"The mean heart rate is preferably around 85; the SD should preferably be higher than 7.5. If the heart rate is higher than 100, record the results and then start a new baseline measurement. If the SD is notably low (< 3) or high (> 20), again note the outcomes and then start a new baseline measurement."

**Group 4 (young adults)**	
Baseline procedure:<br>- During baseline assessment (T0), with 1 min. of walking activity	"The mean heart rate is preferably around 85; the SD should preferably be higher than 7.5. If the heart rate is higher than 100, record the results and then start a new

Settings:<br>- Sensitivity: low<br>- Notifications at: level 4,5<br>- Notifications during: sitting still, walking	baseline measurement. If the SD is notably low (< 3) or high (> 20), again note the outcomes and then start a new baseline measurement."

**Group 5 (adults)**	
Baseline procedure:<br>- During baseline assessment (T0), with 1 min. of walking activity<br><br>Settings:<br>- Sensitivity: low<br>- Notifications at: level 4,5<br>- Notifications during: sitting still, walking	"The mean heart rate is preferably around 85; the SD should preferably be higher than 7.5. If the heart rate is higher than 100, record the results and start a new baseline measurement. If the SD is notably low (< 3) or high (> 20), again note the outcomes and then start a new baseline measurement."

**Group 6 (young adults)**	
Baseline procedure:<br>- During baseline assessment (T0), with 1 min. of walking activity<br><br>Settings:<br>- Sensitivity: low<br>- Notifications at: level 4,5<br>- Notifications during: sitting still, walking	"The mean heart rate should preferably be around 80 (range: 60-90); the SD should preferably be higher than 7.5 (range: 5-12.5). If the heart rate or SD falls outside the range, note the results and start a new baseline measurement, paying close attention to the variation offered. Then choose the results of the baseline measurement that best matches the preferred settings; where the SD is of greater importance than the mean. "

**Group 7 (adults)**	
Baseline procedure:<br>- During baseline assessment (T0), with 1 min. of walking activity<br><br>Settings:<br>- Sensitivity: low<br>- Notifications at: level 4,5<br>- Notifications during: sitting still, walking	"The mean heart rate should preferably be around 80 (range: 60-90); the SD should preferably be higher than 7.5 (range: 5-12.5). If the heart rate or SD falls outside the range, note the results and start a new baseline measurement, paying close attention to the variation offered. Then choose the results of the baseline measurement that best matches the preferred settings; where the SD is of greater importance than the mean."

Supplementary Table 2. Summary of unstandardized parameter estimates (coefficient, standard error, t- and p- test statistics) on SCED outcome measures (two-level analysis).

**Variable**	**Intercept (B0)**				**Time (B1, phase A_1)**				**Time (B1, phase B)**				**Phase B:A_1 (B2)**				**Time x Phase (B3)**			
	Coeff	*SE*	*t*	*p*	*Coeff*	*SE*	*t*	*p*	*Coeff*	*SE*	*t*	*p*	*Coeff*	*SE*	*t*	*p*	*Coeff*	*SE*	*t*	*p*
EMA measures																				
Anger	1.623	.304	5.344	0	-.037	.012	-1.569	.119	.002	.012	.234	.815	-.060	.271	-.221	.825	.040	.027	1.462	.145
Aggressive thoughts	1.509	.265	5.701	0	-.055	.036	-1.508	.133	.012	.014	.873	.384	-.075	.326	-.229	.820	.066	.036	1.867	.064
Aggressive behavior	1.256	.171	7.330	0	-.012	.016	-.744	.458	.003	.006	.420	.675	-.171	.162	-1.053	.294	.014	.016	.891	.374
Behavioral control	4.076	.313	13.022	0	.045	.029	1.537	.126	-.008	.013	-.601	.549	-.048	.362	-.132	.895	-.053	.032	-1.657	.099
Physical tension	2.565	.405	6.328	0	.019	.024	.797	.427	-.009	.013	-.674	.501	-.398	.287	-1.384	.168	-.028	.029	-.988	.325
HR measures																				
Mean	90.767	2.620	34.645	0	.112	.212	.527	.598	.079	.071	1.107	.270	-3.001	2.532	-1.185	.237	-.033	.226	-.147	.884
SD	14.688	1.344	10.928	0	-.094	.100	-.933	.352	.038	.043	.874	.383	1.164	1.069	1.089	.277	.131	.103	1.277	.203

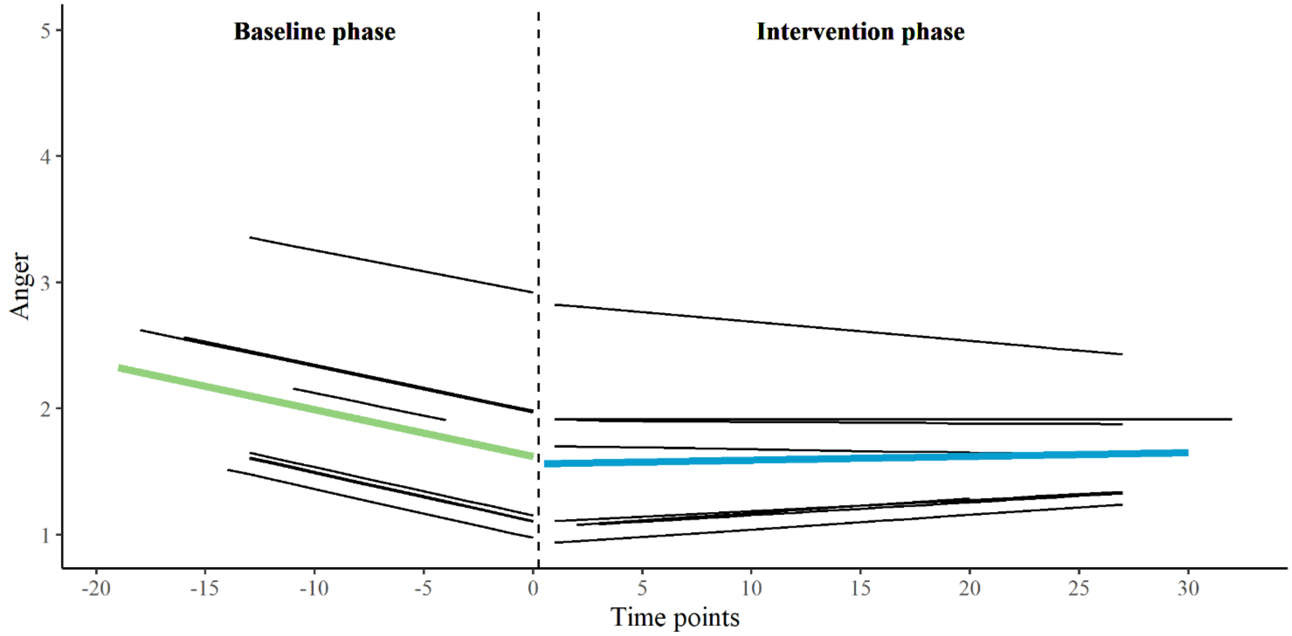

Supplementary Figure 1. Combination of one- and two-level regression results for exploratory EMA measure anger in baseline phase A_1 and intervention phase B.

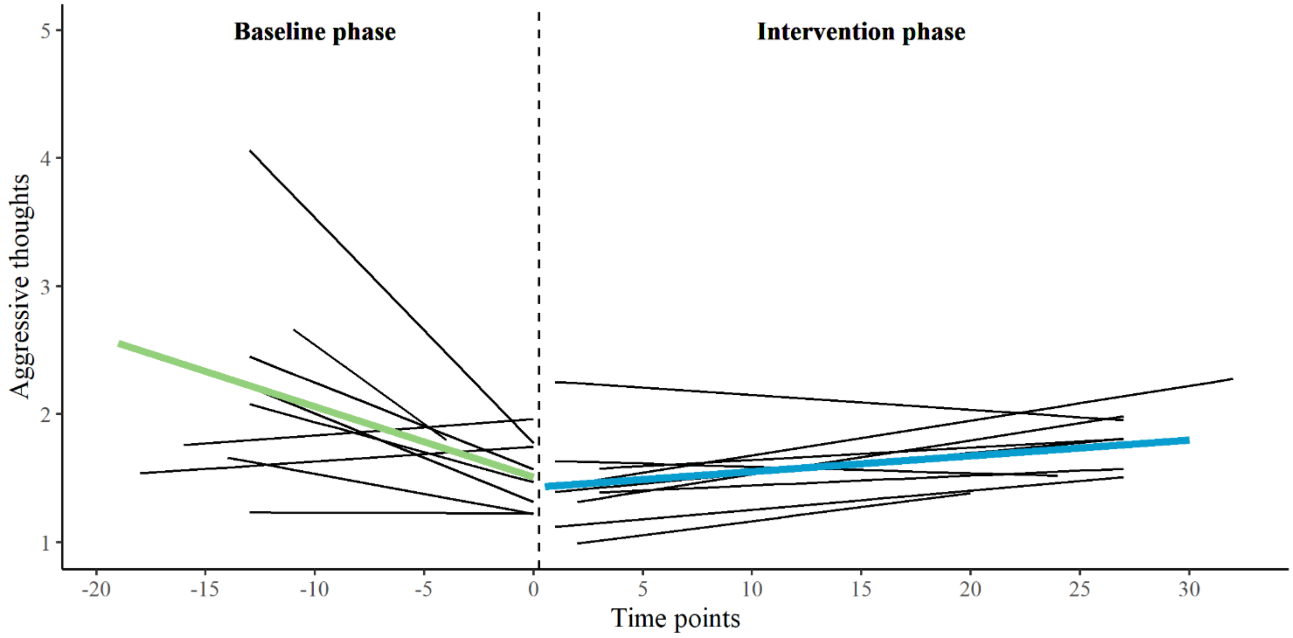

Supplementary Figure 2. Combination of one- and two-level regression results for exploratory EMA measure aggressive thoughts in baseline phase A_1 and intervention phase B.

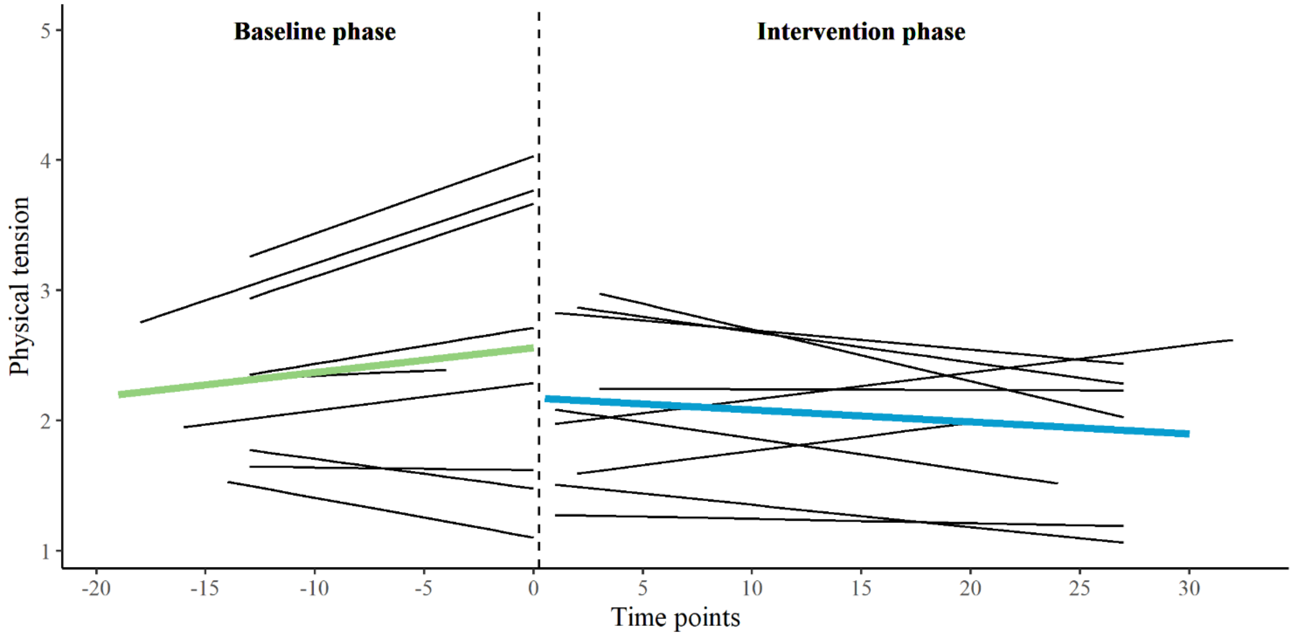

Supplementary Figure 3. Combination of one- and two-level regression results for exploratory EMA measure physical tension in baseline phase A_1 and intervention phase B.

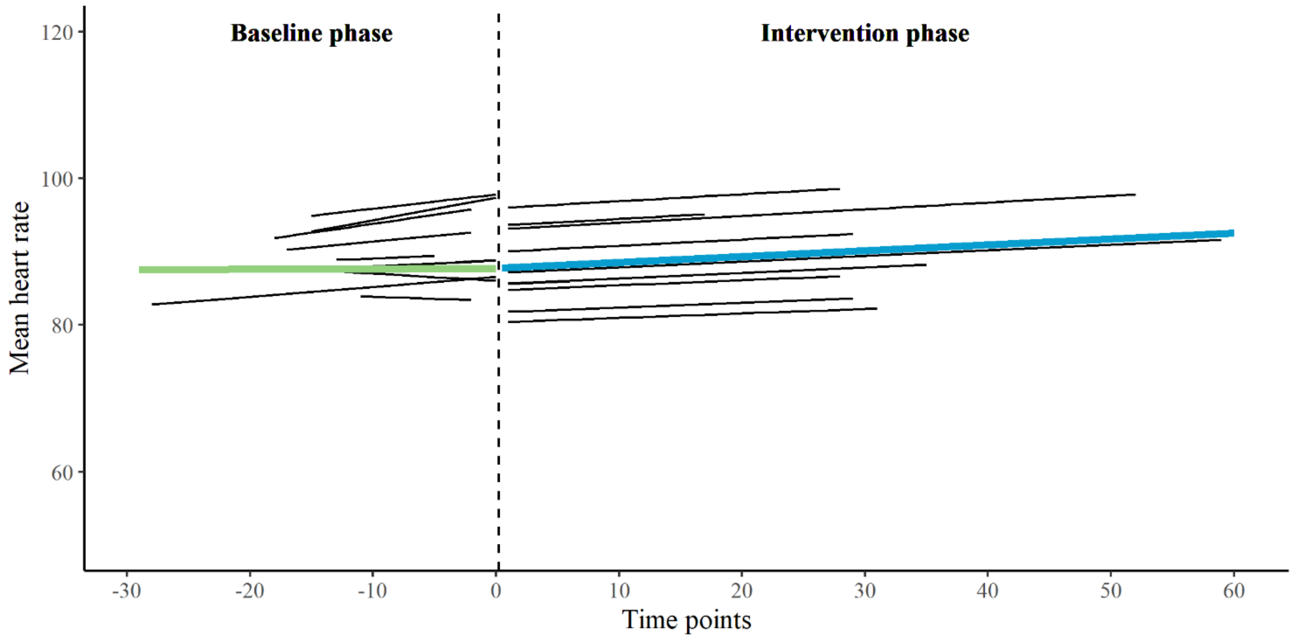

Supplementary Figure 4. Combination of one- and two-level regression results for exploratory measure mean heart rate in baseline phase A_1 and intervention phase B.

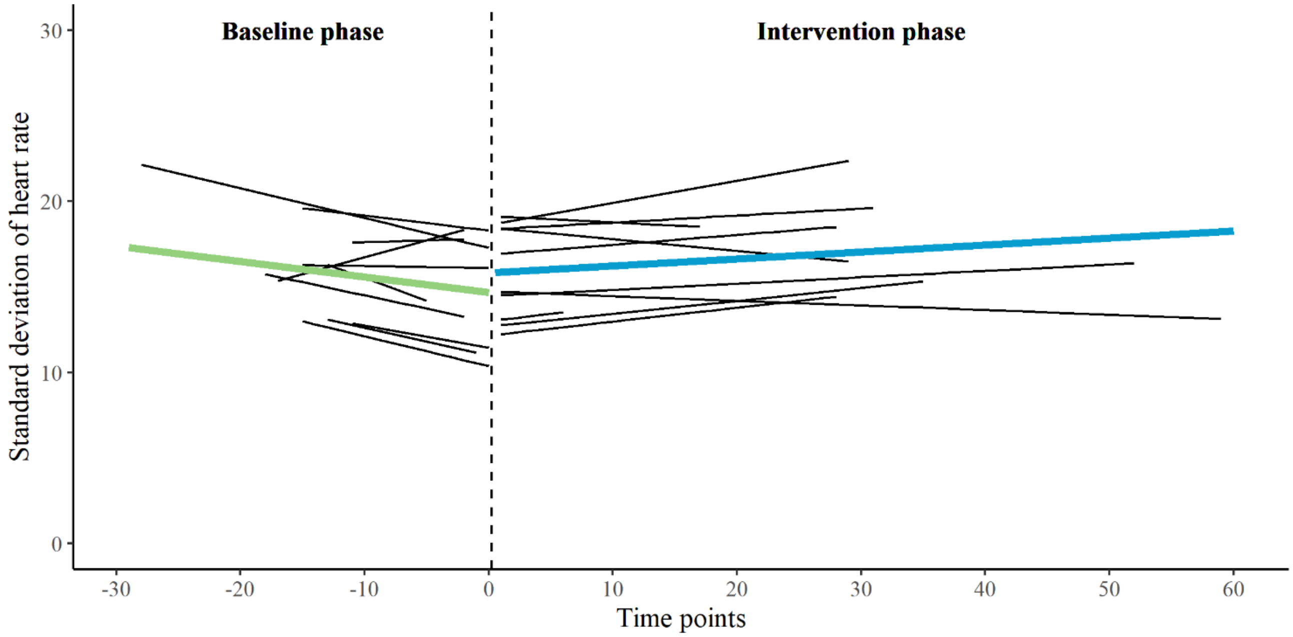

Supplementary Figure 5. Combination of one- and two-level regression results for exploratory measure standard deviation of heart rate in baseline phase A_1 and intervention phase B.

Supplementary results section (one-level piecewise regression analyses)

First, we investigated the developmental effects in the baseline phase. Regarding aggressive behavior, an increase was found for one participant (*B1* = .26, *SE* = .087, *t* = 2.975, *p* = .009). In another participants, aggressive thoughts decreased during the baseline phase (*B1* = -.119, *SE* = .041, *t* = -2.9, *p* = .007). For all other participants and on all other EMA and HR variables, no significant in- or decreases were found for the baseline phase.

Second, we studied developmental effects in the intervention phase. For aggressive thoughts, an increase was found for one participant (*B1* = .044, *SE* = .017, *t* = 2.505, *p* = .009). For all other participants and on all other EMA and HR variables, no significant in- or decreases were found for the intervention phase.

Third, we studied the immediate changes when transitioning into the intervention phase. In one participant, results indicated an immediate decrease in anger at the start of the intervention phase (*B2* = -2.597, *SE* = .792, *t* = -3.28, *p* = .005). For all other participants, as well as on all other EMA and HR variables, no significant effects were found.

Fourth, we studied the interaction effect for time with phase for all variables. Regarding aggressive thoughts, one participant showed a strong increase in the intervention phase compared to a strong decrease in the baseline phase (*B3* = .162, *SE* = .045, *t* = 3.651, *p* = .001). Aggressive behavior slightly decreased in the intervention phase compared to a strong increase in the baseline phase for one participant (*B3* = -.282, *SE* = .089, *t* = -3.169, *p* = .006). For HR-related measures, we found a decrease in mean HR in the intervention phase compared to an increase in the baseline phase for one participant (*B3* = -1.957, *SE* = .582, *t* = -3.365, *p* = .007). For all other participants, as well as on all other EMA and HR measures, no significant interaction effects were found between time and phase.
